# Findings From RAISE Act Research: Family Caregiver Priorities

**DOI:** 10.1093/geroni/igab046.243

**Published:** 2021-12-17

**Authors:** Pamela Nadash, Eileen Tell, Carol Regan, Taylor Jansen, Andrew Alberth, Marc Cohen

**Affiliations:** 1 University of Massachusetts Boston, University of Massachusetts Boston, Massachusetts, United States; 2 ET Consulting, Belmont, Massachusetts, United States; 3 Community Catalyst, Boston, Massachusetts, United States; 4 UMass Boston, Boston, Massachusetts, United States; 5 University of Massachusetts Boston, Boston, Massachusetts, United States

## Abstract

To understand the needs and policy priorities of family caregivers, the Advisory Council commissioned research: first, through a request for information (RFI) in the Federal Register, which garnered roughly 1600 responses. Qualitative analysis revealed that family caregivers have diverse needs spanning their financial security as well as their needs for caregiver-focused supports; recommendations were similarly diverse, including requests for caregiver pay, improved access to respite, and other major policy changes. These findings fed into 12 focus groups focusing on diverse populations of caregivers, yielding more depth around caregiver priorities. Six stakeholder listening sessions built on these results, aiming to develop concrete suggestions for a national caregiver strategy – a key outcome of the Advisory Council. Such strategies ranged from a major publicity campaign creating awareness of family caregivers, to suggestions on implementing caregiver assessments, to more ambitious goals such as improved financing for long term services and supports more broadly.

